# The Relationship between Prevention and Treatment of Colorectal Cancer and Cancerous Toxin Pathogenesis Theory Basing on Gut Microbiota

**DOI:** 10.1155/2020/7162545

**Published:** 2020-02-19

**Authors:** Tianqing Sang, Wenli Qiu, Wenting Li, Hongli Zhou, Haibin Chen, Hongguang Zhou

**Affiliations:** ^1^Department of Oncology, Affiliated Hospital of Nanjing University of Chinese Medicine, Nanjing 210029, Jiangsu Province, China; ^2^Department of Radiology, Affiliated Hospital of Nanjing University of Chinese Medicine, Nanjing 210029, Jiangsu Province, China; ^3^Institute of Oncology, The First Clinical Medical College, Jiangsu Collaborative Innovation Center of Traditional Chinese Medicine Prevention and Treatment of Tumor, Nanjing University of Chinese Medicine, Nanjing 210046, Jiangsu Province, China; ^4^Liaoning University of Chinese Medicine, Shenyang 110847, Liaoning Province, China; ^5^Science and Technology Department, Jiangsu Collaborative Innovation Center of Traditional Chinese Medicine Prevention and Treatment of Tumor, Nanjing University of Chinese Medicine, Nanjing 210046, Jiangsu Province, China

## Abstract

Gut microbiota is a diverse consortium of bacteria, fungi, protozoa, and viruses in the gut of all mammals. Gut microbiota remains in steady state under normal conditions. Changes in the internal and external environment may cause gut Microbiota to be out of tune. Malignant tumors are one of the major diseases currently endangering human health. CRC (colorectal cancer) has a significant upward trend in morbidity and mortality in many parts of the world. Technological advances have not yet brought about a breakthrough in the efficacy of CRC. The development of colon cancer is closely related to gut microbiota imbalance. According to more than 60 years of clinical practice, Professor Zhongying Zhou first proposed the pathogenesis theory of “cancerous toxin” in the 1990s and believed that cancerous toxin was a key pathogenesis of tumor development. Under the guidance of the theory of cancerous toxin, combined with clinical practice, Professor Zhou created an effective anticancer Chinese herbal compound, Jiedu Xiaoai Prescription. This paper summarizes recent hotspots related to gut microbiota and the occurrence, development, and prevention of colon cancer at home and abroad. The relationship between gut microbiota and cancerous toxin theory is proposed, and the feasibility of further studying the biological basis of cancerous toxin pathogenesis theory from the perspective of gut microbiota is pointed out.

## 1. Introduction

Malignant tumors are one of the major diseases that seriously endanger human health. Research on the pathogenesis and treatment of tumors has always been a hot topic in medical research [1, 2]. Technological advances have not yet brought about a breakthrough in the efficacy of cancer. The difficulty and cost of prevention and treatment of tumors continue to increase, and the current treatment of malignant tumors remains a cosmopolitan problem [3]. The antitumor treatment of Traditional Chinese Medicine (TCM) has undergone thousands of years of clinical practice, and its efficacy is unquestionable. It is an important part of comprehensive treatment of malignant tumors [4–6]. Colorectal cancer (CRC) is the second most common malignant tumor in women and the third most common one in men which has high morbidity and mortality [7]. In 2012, there were nearly 1.4 million new cases of colorectal cancer worldwide and 694,000 deaths [8]. Recently, the incidence and mortality of CRC have increased in China [9]. Reducing the incidence of CRC and improving the survival rate and quality of life of CRC patients has become an urgent problem to be solved. TCM treatment has been used for the diagnosis and treatment of CRC for a long time. It is guided by the theory of TCM and adopts the syndrome differentiation treatment method. It plays an important role in interfering with precancerous lesions, attenuating side effects and increasing efficiency, resisting metastasis and recurrence, helping patients survive with tumors, and improving the quality of their lives through Traditional Chinese Medicine compounds (TCD) [10, 11]. The anti-CRC effect of TCM therapy has received more and more attention and recognition from the international community. However, there are still many problems in the TCM treatment of CRC. The core problem is modern research on innovative TCM theory and effective antitumor prescription under the guidance of this theory is not enough. TCD and TCM theory embodies the wisdom of history, and it contains profound scientific connotations. It is urgent to interpret it clearly in modern scientific languages [12, 13]. The development and improvement of TCM requires the reference of new ideas, methods, and techniques. Using modern techniques and methods such as system biology, bioinformatics, and microbiology to interpret the scientific connotation of TCM innovation theory, reveal the antitumor mechanism of TCD, and provide scientific basis for clinical promotion of TCM is urgent needed to improve the cure rate of malignant tumors and realize the modernization and internationalization of TCM [14–16].

Cancerous toxin pathogenesis theory is an innovative TCM pathology theory proposed by Professor Zhongying Zhou based on clinical practice for more than 60 years. The theory holds that cancerous toxin is the key to tumorigenesis and is a special pathology produced in the body during tumor pathogenesis [17], which compounded by multiple substances and multiple factors. Cancerous toxin pathogenesis theory has unique theoretical and clinical significance in the treatment of cancer. Under the guidance of this theory, the treatment of cancer is based on “cancer elimination and detoxification,” and an effective anticancer prescription for treating malignant tumors is formed during clinical accumulation, Jiedu Xiaoai Prescription. The ancient Chinese medicine book “Zhongzang Jing” recorded “the incidence of carbuncle is due to Zang-Fu (Viscera) poisoning, not just because of the congestion of the body surface.” It can be seen that ancient Chinese medicine practitioners have realized cancer has the characteristics of difficult cementation, occulted, and invasiveness, which coincides with the cancerous toxin pathogenesis theory proposed by Professor Zhou.

In this paper, we explored the relationship between CRC prevention and cancerous toxin pathogenesis theory from the perspective of gut microbiota, and we aim to provide guidance for further clinical and experimental research.

## 2. The Occurrence, Development, and Prevention of Colorectal Cancer Are Closely Related to Gut Microbiota

### 2.1. Intestinal Flora Participates in the Development of Colorectal Cancer

In human microecology, the gut microbiota is a complex and diverse and extremely active microecological system composed of bacteria, fungi, and viruses in the gut of all mammals [18]. They colonize the intestines to form a relatively stable community and form a dynamic balance with the body that is interdependent, mutually beneficial, coordinated, and mutually constrained and participates in the physiological processes of digestion, absorption, metabolism, nutrition, antagonism, and immunity of the body. They are another “hidden organ,” which is regarded as the “eighth largest organ” of the human body, carrying the “second genome” that controls human health [19–21]. Gut microbiota affects the human body through energy absorption, intestinal permeability, short-chain fatty acids, and choline metabolism, affecting the development of human health and disease [22]. Gut microbiota plays an important role in the occurrence and development of CRC through metabolic ability and its derivatives, which affect the body's immunity, inflammation, and tumor microenvironment [23, 24]. The incidence of cancer in the colon is 12 times higher than that in the small intestine, and the level of bacteria in the colon is one million times higher than that in the small intestine [25, 26]. Studies have shown that a variety of intestinal bacteria are closely related to the development of CRC (Table 1). Inactivation of the inflammatory pathway caused by gut microbiota imbalance promotes the development of CRC [39]. The release of a large number of bacterial toxins caused by imbalance of gut microbiota promotes the development of CRC [40]. For example, F. nucleatum promotes the development of colorectal cancer through its FadA adhesin by regulating E-cadherin/*β*-catenin signaling [41]. E. coli strains adheres to and invades CRC cells through the EAE(encoding the bacterial adhesion protein intimin) adhesins [42]. The imbalance of gut microbiota leads to changes in bacterial metabolic capacity, and changes in bacterial metabolism promote the development of CRC [43, 44]. These indicate that the development of CRC is closely related to gut microbiota.

### 2.2. The Role of Gut Microbiota in the Prevention and Treatment of Colorectal Cancer

Gut microbiota plays an important role in the prevention and treatment of CRC while participating in the promotion of CRC development. After the rats were given *Bifidobacterium longum*, the precancerous lesions of the colon (ileocecal recess abnormal lesions) decreased significantly [45]. The antitumor effect of probiotics is mainly related to the regulation of intestinal flora, enhancement of immunity, and direct inhibition of tumor-related molecules. Cell research shows that probiotics can indeed affect the proliferation, apoptosis, and adhesion of colon cancer cells [46]. Studies have shown that a variety of intestinal bacteria are closely related to the CRC development (Table 1).

Gut microbiota can also increase the efficacy of chemotherapeutic drugs and reduce the side effects of antitumor treatment. Commonly used anti-CRC chemotherapeutic drugs include cyclophosphamide (CTX), 5-fluorouracil (5-Fu), and irinotecan (CPT-11). Their anti-CRC effects are related to the significant influence on the composition of intestinal microecology. Gut microbiota plays an important role in various CRC treatments such as chemotherapy, immunotherapy, and laparoscopic radical surgery. In 2013, science reported that the efficacy of antitumor immunotherapy and platinum-based chemotherapy was reduced in mice with intestinal flora imbalance, and the presence of intestinal bacteria helped activate antitumor inflammatory responses [47]. Inhibition of intestinal Gram-positive cocci in mice reduces the antitumor effect of CTX [48]. CTX exerts antitumor effects by altering the gut microbiota, inducing bacteria into secondary lymphoid organs, stimulating Th17 cells and Th1 cells to produce an immune response, Also, this effect is not effective in sterile mice and mice that kill Gram-negative bacteria with antibiotics [48]. The initial gut microbiota composition is the key factor driving 5-FU to exert an antitumor effect [49]. Gut microbiota can affect the development of toxic side effects during CPT-11/5-FU treatment, and the use of dietary fiber has the potential to reduce the toxicity of CPT-11 [50]. The gut microbiota affects the antitumor effect of tumor immunosuppressive therapy (ICT) by regulating the host's immune response [47]. The study of gut microbiota changes in CRC patients before and after laparoscopic radical surgery showed that the diversity and abundance of normal human gut microbiota were higher than those of CRC patients, which was related to the age and clinical stage of the patients, and the analysis of gut microbiota helps guide clinical treatment [51]. These indicated that the prevention and treatment of CRC is closely related to the gut microbiota.

## 3. Intestinal Flora Provides a New Target for the Prevention and Treatment of Colorectal Cancer by TCM

Gut microbiota activates the relevant signaling pathway by direct contact with the tumor; affects host genomic stability by causing intestinal mucosal inflammatory responses and immune responses; regulates tumor proliferation, angiogenesis, metastasis, and drug resistance through host metabolites; and mediates immune escape by negative regulation of immune cells and immunological negative regulators. Gut microbiota affects the efficacy of chemotherapy and immunotherapy by regulating various complex pathways such as local microenvironment and signal-regulating protein and plays an important role in the development and prevention of CRC. The TCM formula is the main treatment method of TCM. The TCM formula exerts its therapeutic effect by drinking from the mouth and absorbed through the digestive tract. It is directly in contact with gut microbiota, and there must be a close relationship between them. Gut microbiota participates in the metabolism of TCM drugs. TCM regulates the community composition of gut microbiota through natural compounds and converts metabolic derivatives of gut microbiota into “progeny molecules” with strong biological activity [52, 53].

The anti-CRC effect of TCM is closely related to gut microbiota (Table 2). Enterotoxin-producing *Bacteroides fragilis* (ETBF) is a key pathogen that promotes tumor cell proliferation and promotes CRC progression [61], Dahuang Mudan prescription can significantly inhibit the proliferation of ETBF in vitro [62], and Gegen Qilian prescription can significantly reduce intestinal ETBF content in patients with type 2 diabetes who have dampness-heat syndrome. Intestinal probiotics, *Lactobacillus* and *Bifidobacteria* can inhibit the occurrence and development of CRC from the aspects of inhibiting pathogenic bacteria, improving vitamin metabolism, maintaining intestinal flora balance, improving local microenvironment, enhancing immunity, and enhancing the efficacy of tumor immunotherapy. Sijunzi prescription can significantly increase the abundance of *Lactobacillus* and *Bifidobacteria* and reduce the abundance of *Enterococcus* [63]. Shenling Baizhu powder can increase probiotics, and reduce intestinal endotoxin and inflammatory factors in the intestine of the mice [64]. Sini prescription, improves colon cancer in mice by enhancing intestinal immunity, inhibiting the secretion of proinflammatory factors, downregulating the marker genes of colon cancer, protecting the colonic mucosal barrier, changing the composition of intestinal flora, reducing pathogenic bacteria, and increasing beneficial bacteria [65]. These indicated that the anti-CRC effect of TCM is closely related to gut microbiota.

## 4. Correlation between Intestinal Flora and Cancerous Toxin

### 4.1. Biomolecular Basis of Cancerous Toxin Associated with CRC

CRC is a complex, refractory, systemic disease caused by multiple genes, multiple links, and multiple factors. Modern medicine believes that any biological phenomenon has its biomolecular basis. Combined with the view of system biology, the occurrence and development of CRC can be understood as the interaction, distribution, and composition of a series of related molecular events. Inflammatory cells, chemokines, inflammatory factors, and vascular endothelial growth factor (VEGF) in tumor tissues are closely related to the development of CRC. Research showed the ratio of neutrophils to lymphocytes affects the prognosis of patients with CRC [66]. The CXC chemokine family (CXCL1∼7) signals through chemokine receptors (CXCR) 1∼8, which play a role in promoting or inhibiting cancer, depending on their ability to inhibit or stimulate the immune system. Activation of the CXCR1/CXCR2 pathway and the CXCR4/CXCR7 pathway is associated with tumor aggressiveness and poor prognosis; the CXCR3 and CXCR5 axes play a role in tumor suppression; and common variants encoding the CXC chemokine gene have also been studied as biomarkers of CRC [67]. Inflammatory factor IL-6 promotes CRC progression by inducing deacetylation of FRA1 [68]. High expression of VEGF-A, VEGF-C, VEGFR-2, and VEGFR-3 promotes invasion and metastasis of CRC and leads to poor survival [69].

The cancerous toxin pathogenesis theory is a malignant tumor pathogenesis theory based on TCM. According to more than 60 years of clinical practice, Professor Zhou first proposed the pathogenesis theory of “Cancerous toxin” in the 1990s and believed that cancerous toxin is a key pathogenesis of tumor development. According to cancerous toxin theory, cancerous toxin is composed of a variety of substances and a variety of factors. These factors are also cemented and mutually causal. Therefore, cancerous toxin is not concentrated in a certain organ, and is unlikely to be a single specific substance. Previous studies have confirmed that the antitumor effect of xiaoai jiedu recipe (JXR) and antipain effect of aitongping capsule (APC) under the guidance of cancerous toxin pathogenesis theory are related to a large number of proteins, genes, and multiple signaling pathways, showing multilink and multitarget antitumor effects. Previous studies have shown ①the mechanism of antihepatocarcinoma of JXR is closely related to the upstream receptors of the TLRs/NF-*κ*B signaling pathway (TLR2, TLR4), intermediate key factors (NF-*κ*B, myeloid differentiation factor 88 (MyD88), tumor necrosis factor receptor-related factor-6 (TRAF-6)), and a large number of downstream factors (hypoxia-inducible factor-1a (HIF-1a mRNA), adhesion molecules (CD44v6), transforming growth factor-*β* (TGF-*β*), cell metalloproteinase 2 (MMP2), IL-6, and VEGF) [70, 71]. ②The mechanism of antipain effect of APC is closely related to reduce the expression of VEGF protein, CXCL12, and its receptor CXCR4 in tumor tissues; reduce NO content in the hypothalamus, pituitary, and lumbar spinal cord; increase *β*-endorphin (*β*-EP) in peripheral blood and decrease 5-hydroxytryptamine (5-HT) and prostaglandin E2 (PGE2) in serum; inhibit cyclo-oxygenase (COX) activity, spinal necrosis factor (TNF)-*α*), and IL-1 expression; and reduce c-fos and SP release [72].

### 4.2. Biomolecular Basis of Intestinal Flora Associated with CRC

As the “second genome” of the human body, the gut microbiota is inevitably involved in a series of molecular events closely related to the occurrence, development, and prevention of CRC, involving a large number of inflammatory cells, chemokines, inflammatory factors, metabolic enzymes, and related signaling pathways in the microenvironment of gut microbiota.

The TLRs/NF-*κ*B signaling pathway is involved in the activation of innate and adaptive immune responses, and the pathway-mediated high expression of chronic inflammatory response factors is closely related to the clinicopathological features of CRC. Related to it, this pathway plays an important role in the development of CRC [73]. Studies have shown that the gut microbiota is associated with this signaling pathway against CRC. Representative research evidence: ①certain gut microbiota bind to TLRs to produce an immune response, induce inflammation, promote cell proliferation, and provide microenvironment for host cells to influence CRC progression [74]. ②Fusarium nucleatum (Fn) has a high degree of adhesion and the ability to invade colonic epithelial cells, and Fn promotes mouse CRC cell proliferation and tumor development by activating this signaling pathway [75]. ③Anaerobic *Streptococcus* interacts with TLR2 and TLR4 in colon cells to increase reactive oxygen species levels and promote cholesterol synthesis and cell proliferation, ultimately leading to colonic dysplasia in CRC mouse models and colonic tumor formation in mice [76]. ④One of the key mechanisms of tumor immunotherapy is to regulate the immune system of the patient through gut microbiota. This process is also closely related to this signaling pathway, and the anti-CRC effect of the probiotic mixture is mediated by increasing TLR2 signaling in the CRC rat model [77].

Gut microbiota is associated with inflammatory cells, chemokines, inflammatory factors, and VEGF and participates in the development of CRC. Representative research evidence: ①clodronate-filled liposomes (CLD) reduced the expression of IL-6, IL-13, IL-10, TGF*β*, and CCL17 and increased the relative abundance of the Firmicutes phylum, especially Clostridiaceae and Lactobacillaceae families in the Azoxymethane(AOM)/Dextran Sulfate Sodium(DSS) mouse model of colon cancer, which produced antitumor effects [30]. ②*Bacteroides fragilis* inhibits CRC by inhibiting the expression of chemokine receptor CCR5 in a murine model of colitis-associated CRC [78]. ③VEGF-C can reduce intestinal inflammation in chronic colitis by enhancing lymphatic drainage, regulating IL-9/IL-17 balance, improving gut microbiota [79].

**Figure 1:**
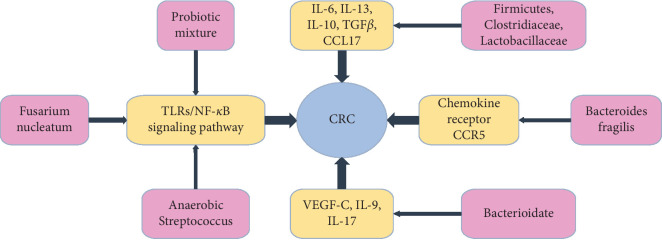
Gut microbiota affects CRC through inflammatory cells, chemokines, inflammatory factors, VEGF, and the TLRs/NF-*κ*B signaling pathway.

The antitumor effect of gut microbiota is closely related to inflammatory cells, chemokines, inflammatory factors, VEGF, and the TLRs/NF-*κ*B signaling pathway (Figure 1). The antitumor effect of JXP and APC under the guidance of cancerous toxin pathogenesis theory are also closely related to these proteins, genes, chemokines, and pathway. These indicate that the cancerous toxin pathogenesis theory and gut microbiota play an important role in the development and prevention of CRC. They are to understand the pathophysiology of tumors from different angles. There must be a certain correlation between the cancerous toxin pathogenesis theory and the gut microbiota.

## 5. Theoretical Study on the Cancerous Toxin Pathogenesis Theory Based on Gut Microbiota

Based on previous studies, the cancerous toxin pathogenesis theory is the guiding ideology. Fecal and peripheral blood of CRC patients and colorectal cancer-bearing mice before and after treatment with JXP/APC are taken as research the object. Modern microbiology, bioinformatics, cell biology, molecular biology, statistical methods, high-throughput sequencing, fluorescent microbead array, and fecal bacterial transplantation technology were used to obtain the composition and changes of the flora in the feces before and after treatment in patients and mice. By studying the compositional differences of the intestinal flora, construct an intestinal flora-key factor regulatory network closely related to cancer pathogenesis and anti-CRC efficacy and verify the relevant intestinal flora, key factors, possible targets, and related signal path. Intestinal flora research was combined with key factors and signal pathway studies, the correlation between cancer and intestinal flora was identified, possible biomarkers were found, and the anti-CRC effect from the intestinal flora network level was revealed. The mechanism further clarifies the biological basis of the theory of cancer pathogenesis and provides a basis for improving the efficacy of CRC.

## 6. Prospects

Recent advances in DNA sequencing technology and computational biology have revolutionized the field of microbiology. With the development and application of powerful bioinformatics analysis tools, it provides better help for in-depth analysis of the significance of the gut microbiota data and the mechanism behind it and provides new ideas for studying the composition and structure of human gut microbiota. There will be more correlations between the gut flora and various diseases (including CRC) being discovered, and gradually identify the specific microbial lineage and related diseases and determine the specific flora of various diseases. The purpose of this paper is to combine the innovative theory of Cancerous toxin pathogenesis with the latest advances in the field of gut microbiota research, gradually integrating metagenomics, macrotranscriptomics, next-generation high-throughput sequencing technology, gene chips, and sterile mice modern techniques. Using the intestinal flora as an entry point to further clarify the biological basis of the cancerous toxin pathogenesis theory, it is helpful to reveal the scientific connotation of the cancerous toxin pathogenesis theory, to provide a theoretical reference for gut microbiota research and colon cancer prevention, and to strive to incorporate these developments into the future of colon cancer clinical practice.

## Figures and Tables

**Table 1:** Gut microbiota associated with CRC.

Bacterial species	Mechanism	Effect on CRC
Escherichia coli	It produced genetic toxins such as colibactin and/or interference leading to DNA mismatch repair (MMR) [27]	Promotes CRC

Peptostreptococcus anaerobius	It interacted with TLR2 and TLR4 in colon cells to increase reactive oxygen species levels and promote cholesterol synthesis and cell proliferation [28]	Promotes CRC

Fusobacterium	Fusobacterium and their virulence factors enriched in the intestinal tract of CRC patients [29, 30]	Promotes CRC

Providencia	Providencia and their virulence factors enriched in the intestinal tract of CRC patients [29, 31]	Promotes CRC

Bacteroides	A positive correlation was observed between the abundance of Bacteroides species and the CRC disease state [31, 32]	Promotes CRC

Bifidobacterium adolescentis	It inhibited the proliferation of LT-29, SW480, and Caco2 colon cancer cells and alters their cell morphology [33]	Anti-CRC

Lactobacillus	Lactic acid bacteria-derived polyphosphate phosphate induced apoptosis in colon cancer cells [34, 35]	Anti-CRC

Bacillus	Bacillus polysaccharide adhered to the surface of colonic adenocarcinoma cells [36]	Anti-CRC

Bulgarian bacilli	Increased abundance of Bulgarian bacilli inhibited the development of CRC [37]	Anti-CRC

Propionibacterium freudenreichii	Propionibacterium supernatants or Propionibacterium metabolites (propionate and acetate) in combination with TRAIL increased proapoptotic gene expression (TRAIL-R2/DR5) and decreased antiapoptotic gene expression in HT29 human colon (FLIP, XIAP) [38]	Anti-CRC

**Table 2:** TCM formula or extract exerts anti-CRC effect based on gut microbiota.

TCM formula or extract	Bacterial species	Mechanism
Isoliquiritigenin	Escherichia, Enterococcus, and some probiotics (Butyricicoccus, Clostridium, and Ruminococcus)	It reduced the abundance of opportunistic pathogens and increased the levels of probiotics [54]

Ginsenosides Rb3 and rd	Bifidobacterium, Lactobacillus, Bacteroides acidifaciens, Bacteroides xylanisolvens, Dysgonomonas, and Helicobacter	It exerted anticancer effects by holistically reinstating mucosal architecture, improving mucosal immunity, promoting beneficial bacteria, and downregulating cancer-cachexia-associated bacteria [55]

Black raspberry anthocyanins	Eubacterium rectale, Faecalibacterium prausnitzii, Lactobacillus Desulfovibrio, and Enterococcus	It modulated the composition of gut commensal microbiota and changed in inflammation, and the methylation status of the SFRP2 gene may play a central role in the chemoprevention of CRC [56]

Epigallocatechin gallate	Bacteroides, Clostridiaceae, Ruminococcus, Bifidobacterium, and Lactobacillu	It maintained a relatively stable structure of the gut microbiota and enriched probiotics [57]

Hydrolysed inulin	Lactobacillus, Bifidobacteria, Escherichia coli, and Salmonella enterica serovar typhi	It altered selected intestinal microbiota to alleviate the azoxymethane-induced preneoplastic aberrant crypt foci in Sprague Dawley rats [58]

Glycyrrhiza polysaccharide	Enterorhabdus, Odoribacter, Ruminococcaceae_UCG_014, enterococcus, Ruminococcaceae_UCG_010, Ruminiclostridium, Parasutterella, and Clostridium sensu stricto	It exerted anticancer effects by reducing opportunistic pathogens and enriching probiotics [59]

Gegen qinlian decoction	Bacteroides acidifaciens, Anaeroplasma, and Bacteroidales	Gegen qinlian decoction enhanced the effect of PD-1 blockade in colorectal cancer with microsatellite stability by remodelling the gut microbiota and the tumour microenvironment [60]
